# Differences between high- and low-achieving pre-clinical medical students: a qualitative instrumental case study from a theory of action perspective

**DOI:** 10.1080/07853890.2021.1967440

**Published:** 2022-01-12

**Authors:** Chan Choong Foong, Nur Liyana Bashir Ghouse, An Jie Lye, Vinod Pallath, Wei-Han Hong, Jamuna Vadivelu

**Affiliations:** Medical Education and Research Development Unit (MERDU), Faculty of Medicine, Universiti Malaya, Kuala Lumpur, Malaysia

**Keywords:** Qualitative instrumental case study, theory of action, academic achievement, high-achieving, low-achieving

## Abstract

**Background:**

Poor academic performance and failure can cause undesired effects for students, schools, and society. Understanding why some students fail while their peers succeed is important to enhance student performance. Therefore, this study explores the differences in the learning process between high- and low-achieving pre-clinical medical students from a theory of action perspective.

**Methods:**

This study employed a qualitative instrumental case study design intended to compare two groups of students—high-achieving students (*n* = 14) and low-achieving students (*n* = 5), enrolled in pre-clinical medical studies at the Universiti Malaya, Malaysia. Data were collected through reflective journals and semi-structured interviews. Regarding journaling, participants were required to recall their learning experiences of the previous academic year. Two analysts coded the data and then compared the codes of high- and low-achieving students. The third analyst reviewed the codes. Themes were identified iteratively, working towards comparing the learning processes of high- and low-achieving students.

**Results:**

Data analysis revealed four themes—motivation and expectation, study methods, self-management, and flexibility of mindset. First, high-achieving students were more motivated and had higher academic expectations than low-achieving students. Second, high-achieving students adopted study planning and deep learning approaches, whereas low-achieving students adopted superficial learning approaches. Third, in contrast to low-achieving students, high-achieving students exhibited better time management and studied consistently. Finally, high-achieving students proactively sought external support and made changes to overcome challenges. In contrast, low-achieving students were less resilient and tended to avoid challenges.

**Conclusion:**

Based on the theory of action, high-achieving students utilize positive governing variables, whereas low-achieving students are driven by negative governing variables. Hence, governing variable-based remediation is needed to help low-achieving students interrogate the motives behind their actions and realign positive governing variables, actions, and intended outcomes.

**Key Messages**
- This study found four themes describing the differences between high- and low-achieving pre-clinical medical students: motivation and expectation, study methods, self-management, and flexibility of mindset.
- Based on the theory of action approach, high-achieving pre-clinical medical students are fundamentally different from their low-achieving peers in terms of their governing variables, with the positive governing variables likely to have guided them to act in a manner beneficial to and facilitating desirable academic performance.
- Governing variable-based remediation may help students interrogate the motives of their actions.

## Introduction

Poor academic performance and failure are unfavourable outcomes that should be addressed as early as possible [1–3]. A recent systematic review of international medical schools reported an attrition rate ranging between 2.4% and 26.2%, making for an average of 11.1% [4]. Meanwhile, a Malaysian study reported that 2.1% to 12.1% (mean 5.3%) of Year 1 medical students enrolled at a public medical school failed to progress to the next stage of their studies [5]. This situation is of particular concern insofar as public medical school training is substantially subsidized by the Malaysian government, with poor outcomes constituting a financial loss in a developing country. This expense is compounded by the fact that remediation for low-achieving students requires substantial time and resources from the educational institution. Moreover, low-achieving students in Malaysia have been found to suffer stress and depression [6,7]. As such, it is vital that medical educators guide students to excel to the best of their abilities.

A comparison of high- and low-achieving medical students may elucidate effective and ineffective learning processes as these students represent the extreme ends of academic achievement [8–10]. Several quantitative studies [3,8,11–13] have sought to determine the factors that impact academic output by differentiating between high- and low-achieving students. Such research indicates that low-achieving students tend to demonstrate lower task value, self-efficacy, and self-discipline, and endure greater degrees of anxiety, frustration, and boredom. However, an in-depth qualitative investigation into the learning process—that is, a study employing multiple dimensions of how and why learning takes place [14]—is required to explain the differences between high- and low-achieving students in this respect.

Some scholars have qualitatively examined academic failure [15] and academic success [16]. Their studies found that high-achieving students had high self-expectations, practiced deep learning approaches, and learned from their mistakes [16]. In contrast, low-achieving students tended to set inappropriate self-expectations, adopt ineffective study methods, and normalize failure [15]. However, different institutions have different admission criteria, curricula, teaching and learning activities, and forms of assessment—extraneous variables that may impact students’ academic achievement [17]. Although the influence of extraneous variables can be mitigated by examining learning outcomes at the same institution, few qualitative studies have pursued this approach to exploring the differences between high- and low-achieving students. Moreover, qualitative studies that have examined students within a single institution, such as Todres et al. [9] and Mirza and Usmani [10], have focussed on intermediate and final-year students. Understanding the experiences of students in the early stages of their studies is important for elucidating early remediation [1,18], which can prevent cycles of failure [18,19]. Therefore, after recognizing the gaps in the literature and realizing that some students from our medical school fail while their peers succeed, we decided to explore the differences between the learning processes of high-and low-achieving pre-clinical medical students at the Universiti Malaya, Malaysia. Accordingly, the following research question guided this study:

How did the learning processes between high- and low-achieving students differ?

## Methods

### Study design and context

In order to conduct an in-depth exploration of the learning processes of high- and low-achieving students, this study adopted a qualitative instrumental case study design [20]. More specifically, it constructed and compared two cases: high- and low-achieving students. A qualitative instrumental case study design was utilized because both cases demonstrate very distinct representations (i.e. outstanding achievement and marked failure) [21]. Comparing these cases is intended to determine both effective and ineffective learning processes [22].

This study was conducted at the Universiti Malaya, the oldest and highest-ranking public university in Malaysia, from September 2019 to July 2020. Ethical approval was obtained from the Universiti Malaya Research Ethics Committee (UM.TNC2/UMREC − 647), and written consent forms were obtained from all participants prior to the study.

In Malaysia, medical education entails a five-year undergraduate program. Top performing high school graduates (i.e. A-Level equivalent) typically enter medical school at the age of 19 or 20. Medical graduates are required to undergo mandatory two-year internships at public hospitals before obtaining their licences to practice medicine unsupervised. Gaining acceptance into the medical program is highly competitive. For admission to the Universiti Malaya, applicants are required to possess excellent high school examination results and perform well in both the BioMedical Admissions Test (BMAT) and multiple mini-interviews. Accordingly, the annual intake is approximately only 160 students, the majority of whom are 19 years old. The medical curriculum consists of two stages: a two-year pre-clinical medical stage and a three-year clinical stage. Additionally, curriculum content is horizontally and vertically integrated [23]. Year 1 and Year 2 pre-clinical medical students share similar education environments, such as didactic lectures, interactive seminars, weekly clinical immersion programs, and problem-based learning sessions, whereas, Years 3, 4, and 5 clinical medical students undergo clinical rotations at the teaching hospital.

### Theoretical approach

This study adopts a theory of action approach to examine the differences between high- and low-achieving first-year students at a Malaysian university. According to the theory of action approach, human actions are derived from mental variables that consciously or unconsciously govern respective actions [24,25]. These governing variables can be identified through actions or the rationales given for specific actions [24,25]. Actions are based on these governing variables and result in outcomes. Simply put, governing variables (i.e. “why did I do this?”) decide actions (i.e. “what did I do?”), and the actions decide outcomes (i.e. “what did I get?”).

**Figure 1.**
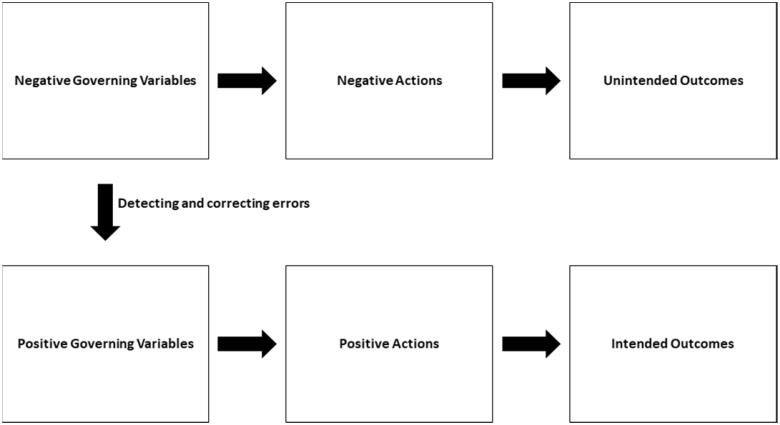
Theory of action [24].

In respect to academic success, the theory of action contends that positive governing variables (i.e. positive values like responsibility) lead to positive actions (e.g. consistency in studying), resulting in intended outcomes (i.e. success). In contrast, negative governing variables (i.e. negative values such as a lack of motivation) lead to negative actions (e.g. procrastination), resulting in unintended outcomes (i.e. failure) (Figure 1). Academic success thus originates from the positive governing variables of high-achieving students, while academic failure is rooted in the negative governing variables of low-achieving students. Intended outcomes can be ensured by altering governing variables, thereby regulating actions [26,27]. As such, investigating the association between governing variables, actions, and outcomes can facilitate the identification and modification of one’s learning process [18].

By identifying the positive governing variables and actions of high-achieving students and correcting the negative governing variables and actions of low-achieving students enrolled in medical education, this study provides insights for the design of interventions intended to enhance academic success among medical students [20].

### Case selection

The selected medical school has pre-clinical assessments on the mastery of basic and clinical sciences knowledge (e.g. a written knowledge-based assessment and anatomy and pathology spot tests), clinical skills (e.g. objective structure clinical examination), public health (e.g. key feature questions), and professionalism (e.g. a reflective portfolio). The written knowledge-based assessment—that is, summative assessment—results are given as a numerical score, whereas the other types of assessment only indicate a satisfactory or unsatisfactory outcome. The written knowledge-based assessment consists of multiple-choice questions examining students’ understanding of normal and pathological human structures, functions, and behaviours relevant to a diagnosis, management plans, and the prevention of health problems in the integrated curriculum (e.g. musculoskeletal sciences, haematology, neurosciences, renal and urology). Students who fail any assessment are required to repeat the entire academic year.

This study selected cases based on assessment results for the 2019/2020 academic year. As students obtained satisfactory results in all assessments except the written knowledge-based assessment, cases were selected based on their performance in the knowledge-based assessment. This selection indicates that high achievement was confined to the cognitive domain. Based on their score in the written knowledge-based assessment, students were invited to a meeting at the beginning of the 2020/2021 year, where the researchers explained the aims, procedures, and measures of the study and ensured participant confidentiality.

Following this process, 31 students who scored in the ninetieth percentile in the written knowledge-based assessment were identified as high-achieving students [28,29] and invited to meet with the researchers, whereafter a total of 14 high-achieving students consented to participate in the study voluntarily. This process was repeated to recruit low-achieving students who scored in the tenth percentile of the knowledge-based assessment [29,30]; however, only 5 of 21 low-achieving students agreed to participate in the study.

### Data collection

We collected data using reflective journals and semi-structured interviews. For the reflective journals, we used a Microsoft Word document template that contained a list of questions, with a text box provided for each question. The questions were designed according to Gibbs’ reflective cycle and intended to encourage participants to reflect on and describe their experiences in the previous academic year [31]. Gibbs’ reflective cycle enables participants to recall and express their actions and feelings and engage in self-reflection; these details provide important insight into the learning process. Content validation was performed with three academicians, followed by face validation and a pilot study with students [31]. Instructions emphasized that there was no right or wrong answer and encouraged students to express their genuine thoughts and feelings. The reflective journal formats for high- and low-achieving students were identical, except that the reflective journal for the former used the phrase “academic success,” while that for the latter used “unsuccessful attempt”; reflective journal formats are provided in Appendix 1. Participants received an empty template of the reflective journal *via* email and returned their completed reflective journals by replying to the sender, thereby ensuring confidentiality. Only the student support officer had access to these emails. All communications between individual students and the student support officer were private. Participants were allocated two weeks to complete their reflective journals.

Following the submission of the reflective journals, semi-structured one-to-one interviews were conducted. Guided by the theory of action approach, interview questions were developed to investigate what (i.e. actions) students did in their previous academic year and why (i.e. governing variables) (Appendix 2). The interviews were conducted in interview rooms at the researchers’ office. The interview rooms were quiet and private. The role of the interviewer was filled by the student support officer at the medical school. She was a medical graduate, who had received in-house training to offer academic performance and well-being support to medical students. The interviewer read the students’ reflective journals prior to the interview in order to obtain a general idea of the students and draft interview prompts. To build a good rapport with students and understand them better, the interviewer asked the students to share their family background and hobbies. Based on students’ initial responses, they were prompted with contingency questions to provide further clarification. Each interview lasted 60 to 80 min. Interviews were audio-recorded and transcribed verbatim, producing a total of 164 pages for high-achieving students and 83 pages for low-achieving students.

Reflective journals and interviews complemented each other. First, important questions were repeated across methods to verify students’ learning approaches (e.g. “what did you do before attending teaching activities?” in the interview and “how did you prepare for the teaching session?” in the reflective journal). Second, the interviews sought further clarification on students’ responses in the reflective journals (e.g. “you mentioned trying to pay attention and writing notes; could you tell me what the process of writing notes involved?”), thereby aligning the reflective journals and interviews.

This study, including all data collection, was conducted in English. English is a compulsory subject in both primary and secondary education in Malaysia and is commonly used in daily communication. English is the medium of instruction in the medical program at the Universiti Malaya, with all students expected to be fluent in both written and spoken English.

### Data analysis

This study employed thematic analysis to examine data. The thematic analysis comprises a set of procedures to identify and describe patterns in the data [32]. This study used this approach because it is flexible and appropriate for studying students’ learning processes from different angles [32,33]. Steps proposed by Braun and Clark [32] were used to analyze interview transcripts and reflective journals. After collecting the data, two analysts read the interview transcripts and reflective journals line-by-line multiple times in order to gain familiarity with the data and achieve contextual sensitivity [21].

Reflective journals and interview transcriptions were saved as Microsoft Word documents and imported into QDA Miner Lite 2.0, a free computer-assisted qualitative analysis software program for analyzing textual data. Using QDA Miner Lite 2.0, two analysts independently searched and coded “what did students do?” and “why did they do this?” for Year 1 high-achieving students. Discussions were held to compare the initial codes and solve discrepancies. Using the agreed-upon codes, the two coders subsequently coded Year 2 high-achieving students. Discussions were held again to verify that there were no newly emergent codes. An identical coding process was applied to analyze Year 1 and Year 2 low achieving students.

Once all data were coded, the two analysts compared the codes of high- and low-achieving students. The two analysts were careful to avoid *a priori* fixed and polarized notions that there will be differences. The two analysts identified eight corresponding patterns; for example, “better time management” among high-achieving students corresponded with “poor time management” among low-achieving students. Subsequently, related patterns were fitted into a theme; for instance, the two patterns of “time management” and “consistency in studies” were categorized under the theme of “self-management.” As participants’ responses in interviews/reflective journals exposed their identities as high- or low-achieving students, the data could not be blinded. Therefore, the third analyst reviewed the coding results to minimize bias. All excerpts were reviewed to ensure that there was no bias in the selection of quotes. The three analysts deduced that data saturation had been achieved as codes were repeated among participants, with low-achieving students noting that they should have taken the actions practiced by high-achieving students [34]. We used both data (reflective journals and interviews) and investigator (analysts) triangulation in this study [35]. Triangulation is conducted to gain an understanding of a phenomenon by using different methods, or data, to examine the convergence of information and minimize bias [36].

## Results

### Descriptive characteristics of participants

A total of 19 pre-clinical medical students participated in this study. These participants consisted of eight Year 1 students and eleven Year 2 students, the majority of whom were either 19 or 20 years old. Their demographics in terms of years of study, age, and gender are summarized in Table 1.

**Table 1.** Participants’ demographics.

	High achieving students	Low achieving students
Year		
1	5	3
2	9	2
Gender		
Male	3	0
Female	11	5
Age		
19	5	3
20	7	2
21	2	0

Data analysis produced four themes: motivation and expectation, study methods, self-management, and flexibility of mindset.

#### Theme 1: Motivation and expectation

##### Intrinsic motivation

High- and low-achieving students exhibited differences in terms of intrinsic motivation and academic expectations. In respect to intrinsic motivation, high-achieving students expressed an interest in medicine, finding the study of the human body and mysteries of medicine fascinating. In contrast, low-achieving students doubted their decision to pursue a medical degree, exhibited little to no interest in medicine, and admitted to feeling lost and aimless in their studies. Low-achieving students also expressed feeling frustrated after observing students from non-medical degree programs have more time to engage in social activities and considered their medical degree a barrier preventing them from enjoying their favourite activities.

##### Academic expectations

In terms of academic expectations, high-achieving students set higher expectations for themselves in terms of academic outcomes, such as aiming to obtain the Dean’s Award and graduating on time. In contrast, low-achieving students had lower expectations and simply wanted to pass their exams. During the interviews, low-achieving students mentioned the need to set higher academic expectations.

#### Theme 2: Study methods

##### Study plan

High- and low-achieving students exhibited differences in their study methods, particularly in terms of planning their studies and their approach to learning. In regard to the former, high-achieving students planned their studies for upcoming weeks, including what they should prepare before attending classes and what to review after classes. By planning ahead, high-achieving students felt secure and calm ahead of classes or exams. In contrast, low-achieving students did not create systematic study plans ahead of time but acted according to their mood at the time. However, low-achieving students noted that they had prepared study plans for the 2020/2021 academic year, which helped them be more organized in their studies compared to the previous year.

##### Learning approach

In respect to the learning approach, high-achieving students attempted to comprehend the medical content by relating different learning topics with one another. For instance, if they learned a pre-clinical topic, they endeavoured to associate it with clinical practices. They thus avoided merely memorizing the medical content without comprehensively understanding and exploring the reasoning behind the body function mechanism. In contrast, low-achieving students adopted superficial learning, typically feeling satisfied with simply skimming or scanning the lecture notes with no clear objective. They also admitted to skipping topics they found less interesting.

#### Theme 3: Self-management

##### Time management

High- and low-achieving students exhibited two differences in terms of self-management, namely, time management and consistency of studying. First, high-achieving students demonstrated better time management than low-achieving students, allocating an appropriate amount of time for studies, friends, family, and relaxation. In contrast, low-achieving students tended to procrastinate studying for examinations until the last minute. However, during the interviews, low-achieving students recognized their lack of time-management skills and were determined to avoid procrastinating going forward.

##### Consistency

In respect to consistency of studying, high-achieving students studied consistently and kept up with the topics discussed in classes. They also repeatedly reviewed their lecture notes to ensure that they were mastering the knowledge and ability to apply it in the future. Although low-achieving students attempted to study consistently, they eventually gave up when the content became challenging. Low-achieving students thus lacked consistency in studying and tended to prioritize enjoying themselves rather than studying.

#### Theme 4: Flexibility of mindset

##### Exposure of vulnerability and support seeking

High- and low-achieving students exhibited two differences in the flexibility of their mindsets, specifically in terms of their exposure to vulnerability and willingness to seek support, and in their willingness to adapt to changes. First, when facing academic and emotional challenges, high-achieving students regularly turned to their peers and family members for support, who encouraged them to strive for better results. In contrast, low-achieving students were reluctant to show weakness to those around them, tending to believe that neither their family members nor peers would understand their struggles and potentially invalidate them. However, during the interviews, low-achieving students claimed to have changed their mindset regarding showing vulnerability and seeking support, acknowledging the importance of reaching out to family, friends, and lecturers when facing challenges. By seeking support from peers and lecturers, low-achieving students found themselves better equipped to face challenges in their studies.

##### Willingness to adapt to changes

With respect to students’ willingness to adapt to change, high-achieving students recalled trying a variety of study methods until they identified the most suitable method for medical studies. In this regard, they noted changing their duration of the study, approach to revision, and use of technology to improve the effectiveness of their studying. In contrast, low-achieving students continued using the same study method despite realizing that it was ineffective. Low-achieving students appeared less resilient and more likely to avoid challenges.

## Discussion

Based on the qualitative data from a small number of students, this study indicated that high-achieving students exhibited greater degrees of motivation and self-expectation, more effective study methods, better self-management, and more flexible mindsets compared to their low-achieving peers. These differences can be explained using a theory of action framework, which contends that positive actions increase the likelihood of attaining favourable academic achievements. This section discusses this study’s theoretical and practical implications, as well as its limitations.

### Theoretical implications

The findings of this study correspond with those of the existing literature. Like previous studies [3,10,16,37], the high-achieving students examined in this study adopted deep learning methods (i.e. methods intended to facilitate an understanding of the content rather than rote memorization) and study consistently. Previous studies also reported that high-achieving students had better class attendance [38], demonstrated better self-discipline when managing their studies [12,13], and showed a willingness to work hard [13]. In addition to using failure as a motivation to improve, high-achieving students tended to learn from their mistakes and were willing to change [3]. They also attributed their academic achievements to the student support received from the medical school [10,16]. The findings of this study also correspond to previous studies demonstrating the relationships between actions and outcomes [8,11,15,39,40]. Although high-achieving students did not use the term “governing variable,” they were likely to be governed by intrinsic motivation [3], a sense of responsibility for themselves and patients, recognition of their own limitations, and open-mindedness. These positive governing variables are fundamental to the intended outcomes for students [25].

**Figure 2.**
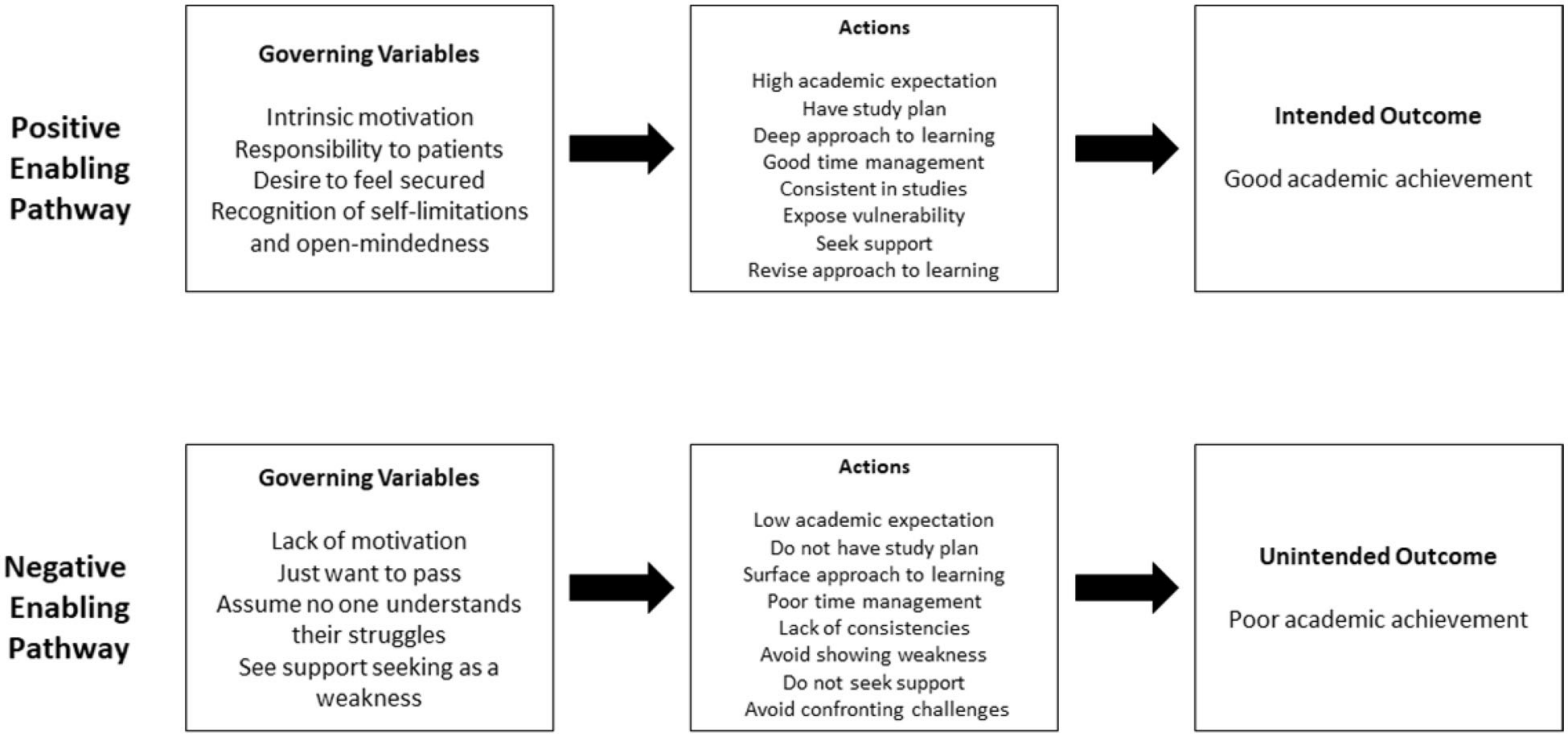
Differences in learning processes between high- and low-achieving students from the Theory of Action perspective.

Correspondingly, negative governing variables may have guided students to act in a way that negatively impacted their academic outcomes [25]. Similar to prior studies, the low-achieving students examined in this study were found to lack concrete motivations to pursue a medical degree and typically “just wanted to pass” [15]. Consequently, these students exhibited low academic expectations [9], adopted a superficial learning approach [15], demonstrated poor time management [15], and did not study consistently [11,41]. Moreover, as they considered needing support a sign of weakness, low-achieving students rarely sought help and tended to avoid confronting challenges [15]. Failure to seek help may result in higher degrees of stress due to an inability to cope with challenges [9,10]. This study found similar negative actions among low-achieving students that led to procrastination and an acceptance of undesirable outcomes [24,25]. Figure 2 illustrates the differences in the learning processes of high- and low-achieving students from a theory of action perspective.

The similarity between the results of this study and those of previous research indicates similar characteristics of high- and low-achieving students across national contexts. Malaysian students are not alone in respect to the challenges of excelling in medical studies. As such, international collaboration and the sharing of resources between medical schools may prove invaluable to address this common issue.

### Practical implications

The findings of this study have important practical implications as they demonstrate the value of effective remediation for early-stage medical students underpinned by a theory of action approach. This result is particularly important insofar as remediation should be extensively mapped into a learning theory to ensure sound design [42]. Indeed, this study actively recruited pre-clinical medical students as participants in order to identify the need for remediation at an early stage of learning. Early remediation is essential to reducing the risk of academic failure and cycles of failure [18].

**Figure 3.**
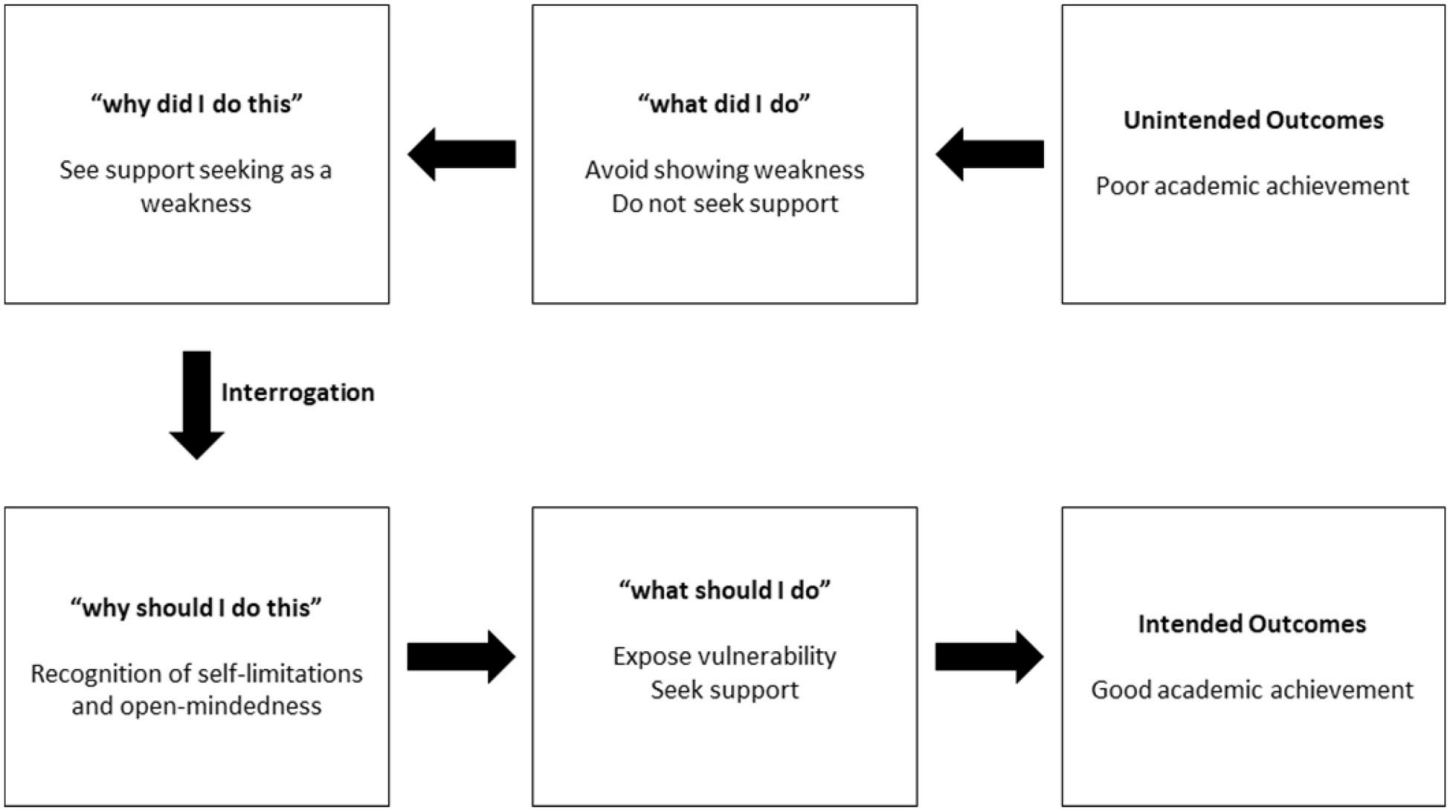
Learning from negative enabling pathway and transforming it to positive enabling pathway.

More specifically, this study proposes governing variable-based remediation. Per Figure 3, this remediation is intended to guide low-achieving students to shift from a Negative Enabling Pathway to a Positive Enabling Pathway. It is imperative that the Negative Enabling Pathway be identified and eliminated. By replacing negative governing variables with positive governing variables, students will be empowered to act proactively and identify positive actions to achieve an intended outcome. Indeed, the appropriate learning approach has been shown to require less effort and lead to better results [24,25], making the proposed remediation more effective. For instance, if possessing positive governing variables, students will actively seek remediation, rather than passively waiting for remediation to be offered. This observation corresponds with the findings of Yip et al. [39] that developing a self-motivated attitude is essential for academic success.

Through governing variable-based remediation, students can be taught to identify their own Negative Enabling Pathways and recognize the misalignment of negative governing variables and intended outcomes. They can subsequently work on developing a Positive Enabling Pathway to realign their governing variables and actions with their intended outcomes [43]. Interrogating one’s governing variables is a process of detecting and correcting errors [24,44,45], one achievable *via* guiding or self-reflection on one’s actions.

In this study, several low-achieving students informed the interviewer that they had made some changes similar to the actions of high-achieving students. Arguably, the process of reflecting on their previous year *via* journaling and the interview helped these students shift from a Negative Enabling Pathway to a Positive Enabling Pathway. However, this postulation remains preliminary and requires further evidence. In this respect, interventions involving the application of governing variable-based remediation are needed to evaluate and further develop the model proposed by this study. Such remediation could provide guided steps to help students identify, reflect on, and correct their governing variables [46]. Guided reflection may be an appropriate first step of governing variable-based remediation, particularly as it can serve as a basis for an enabler diagnostic tool [47] to support students and facilitators in identifying and eliminating negative governing variables. Although the result of unintended outcomes could be influenced by a plethora of factors, governing variable-based remediation will support the development of resilient and lifelong learners who are self-aware and able to adapt to any situation and context.

However, remediation is meaningless if students do not embrace it. In this study, the low achieving students failed to seek help when they needed it. This resistance to seeking help might be due to the effects of learned helplessness [48] and impostor syndrome—soloist [49]. To address their learned helplessness, low-achieving students could recall their experiences of previous successes and accomplishments to strengthen their convictions that further efforts can produce similar successes at medical school [50]. Since soloists see help-seeking as a sign of failure, the governing variable-based remediation must be made non-threatening to students. Furthermore, the remediation should be actively proffered to students.

### Limitations

Our study has several limitations as follows:

- First, this qualitative instrumental case study was conducted at a single institution, indicating that the explanation illustrated in Figure 2 may not be generalizable. A description of the research site helps enhance the transferability of findings in qualitative studies [51]. Accordingly, the findings of this study may be transferrable to other medical schools sharing similar characteristics. Nevertheless, future studies should consider a multi-institutional study with larger samples. This study constitutes an initial step, with future investigations recommended to consolidate and evaluate the model by collecting empirical evidence (Figure 3). Quantitative experimental design or regression analyses may be useful in assessing the cause-and-effect relationship between governing variable-based remediation and academic performance.
- Second, differences in the learning process are not the only explanation for academic success or failure. While the determinants of academic performance are multifactorial [4,40], this study could not investigate all possibilities. Some explanations lie beyond the learning process, including personal, social, family, health, and financial issues [52,53]. In this study, the participants’ socioeconomic backgrounds were not measured; however, this factor could have influenced their academic performance. For example, students whose parents are physicians could have experienced greater financial stability and received more guidance and advice concerning the challenges they might face in medical school. Additionally, further investigations are needed to understand why low-achieving students either are not able to seek help or feel uncomfortable doing it. Therefore, there is a need to identify students at risk of low academic achievement and to provide them with the necessary support.
- Third, while one-time individual interviews were in-depth and practical, this may have condensed discussion of the learning processes that occurred over the year and overlooked some details. Future investigations should consider conducting follow-up interviews with participants.
- Fourth, case selection was confined to cognitive acquisition (i.e., written knowledge-based assessment). The findings of this study do not explain students’ performance differences in other types of assessment, namely, attitudes and skills acquisition. Using similar methods to those employed in this study, future studies should consider investigating the differences in various types of assessment. Such research may prove useful in demonstrating the generalizability of differences between high- and low-achieving students in respect to the learning process.
- Fifth, low-achieving students were less responsive to participating in this study despite receiving the same presentation of the study’s aim and procedures and being assured that all data were confidential. This low participation rate may be attributable to the fact that low-achieving students tend to be unwilling to share their experiences and feelings due to low self-esteem [54]. In improving the participation rate of low-achieving students, future researchers may wish to consider building long-term relationships with students prior to conducting their research, encouraging participation by establishing trust and mutual understanding of how sharing learning experiences may benefit them. In this study, low-achieving students expressed similar comments, indicating redundancy and the interview data are rich. A systematic review of justifying sample size sufficiency in qualitative studies recognizes the aforementioned criteria for data saturation and sample adequacy [55]. Furthermore, interview data were triangulated with reflective journal data. Future qualitative studies encountering low participation rates may wish to adopt the methodology demonstrated in this study to ensure the credibility of their data.
- Finally, to be empathic and ethical, researchers did not request that high- or low-achieving students justify their decision not to participate in the study. Future studies may wish to investigate reasons for refusing to participate in such research, as this could aid in developing a better engagement plan.

## Conclusion

Learning improvements for both high- and low-achieving pre-clinical medical students should be taken into consideration in order to review their governing variables, which, in turn, influence their learning processes. Furthermore, low-achieving pre-clinical medical students must interrogate the motives of their actions and realign their positive governing variables, actions, and intended outcomes.

## Data Availability

The datasets collected and analyzed during this study are available from the corresponding author on reasonable request.

## Appendix 1. Reflective journal

**Table:**

High achieving students:
I would like to thank you for your willingness to participate in this study. There are no right, wrong, desirable or undesirable answers. Feel comfortable expressing what you think and how you feel. Kindly go through and respond to each question. 1. Personal Profile a) Name, date of birth, age, unique or special things about yourself, previous academic experiences, and achievements before entering the university. b) Family background, hometown, parents’ occupation, parents’ academic background, siblings, family members in the medical profession. 2. Reasons for Studying Medicine a) Why did you choose to study medicine? b) Did you have any pre-existing knowledge about the medical profession? If yes, what was the source of this information? c) You had an excellent pre-university result. Why did you not consider choosing other programs? d) What motivated you to continue your studies? e) What kind of doctor do you want to be? 3. Reflection a) i. Which study approach did you use from the start until the end of the academic year? ii. Did you encounter any problem using this study approach? How did you cope with the problem? iii. Elaborate on your study style: • How did you prepare for lectures/teaching sessions? • What was your attendance (%) for lectures/teaching sessions? • Describe your attention/behaviour during lectures/teaching sessions. • What did you do to review the content after lectures/teaching sessions? • How did you manage your studies and social activities on weekends? b) i. How was your social life from the start until the end of the academic year? ii. Did you encounter any problems in your social life? How did you cope with this problem? c) i. How was your personal life/family relationship from the start until the end of the academic year? ii. Did you encounter any problems related to personal life/family relationships? How did you cope with this problem? d) Is there anything else that you would like to share? e) i. Would you consider any of the stated study approaches/styles to have contributed to your academic success? Please justify your answer. ii. What did you think and feel about this study approach/style? Why? iii. What have you learned from that situation? iv. Why do you think that situation happened? v. If you were given a chance to change your action, what would you do? vi. Based on what happened, what is your plan for the current academic year? Describe. vii. How will you make sure that you carry out your action plan properly? Describe. Thank you for your time and effort.
Low achieving students: Note: Identical questions except: Question 3(e)(i), the phrase “academic success” was changed to “unsuccessful attempt.”

## Appendix 2. Interview questions

**Table:**

Prior to commencing the interview, both high- and low-achieving students were: Informed of the purpose of interviews. Given a copy of their consent forms. Confidentiality, anonymity, and the right to withdraw were stated in the consent forms. Asked if they had any questions before the interviews started. After obtaining consent, the interviewer began recording the interviews.
High achieving students:
General questions What happened in the previous academic year? When did you realize that you would score well in the exam? How did you feel after receiving the results? What did you do in the previous academic year? How did you approach learning and studying in your previous academic year? Specific questions What did you do before attending teaching activities such as lectures? (Prompt for specific examples) Why did you do so? (Prompt for reasons) What did you do during the teaching activities? Why did you do so? What did you do after attending the teaching activities? Why did you do so? Hypothetical questions If you could start the academic year over again, which attitude/behavior/action/strategy **would you maintain?** Why? If you had a chance to talk to your juniors, what is the most important piece of advice that you give them in respect to achieving a better performance in their studies? Why?
Low achieving students:
Note: Identical questions except: Question 1, the phrase “score well” was changed to “fail or score poorly.” Question 3, the phrase “would you maintain” was changed to “would you change.”

## Appendix 3. Quotes

**Table:**

No.	Domain	Theme	Sub-theme	Representative quotes	
				High-achieving students	Low-achieving students
1	Non-academic	Motivation and expectation	Intrinsic motivation	High achieving student, HA2, reflective journal: “I chose medicine mainly due to my interest inhuman body. I always find it fascinating at how complex and unique a human body is compared to other animals.”	Low achieving student, LA1, Interview: “I considered switching to another course because I have better creativity than logical reasoning skill. This thought has been hidden in my mind for a long time. It may be architecture, drawing, or designing. I did not feel I belong here. I felt lost. I did not even know why I was in the class, I did not want to be there and I just wanted to get out as soon as I could.” LA1, Interview: “I was in a dilemma whether to continue in medical school. I did not understand why I could not have free time whereas my friends from other courses were having the time of their lives. Life in medical school was different from what I had imagined before I entered it. I thought I could spend more time with people and do my favourites. On Instagram, I could see engineering and architecture students were enjoying their lives but all we could do was studying endlessly.”
			Academic Expectation	HA2, Interview: “I had a target for myself that I wanted to get Dean’s award… I had an aim. For every exam that I sit, I try to score the best that I can, not to ‘beat’ anybody but to improve myself. It is a competition against myself to achieve the highest target.”	LA2, Reflective Journal: “I just wanted to pass the examination and did the bare minimum to get through it. I had the bare minimum mindset.” LA1, Reflective Journal: “I don’t know if this is a high expectation but I want to score as high as possible and I want to get the Dean’s award. It seems to be an ambitious goal since I failed the first year but I want to get into the Dean’s list.”
2	Academic	Study methods	Study plan	HA4, Interview: “After the lectures, I planned what I should do. I read the lecture notes and made my notes. I had a plan so that I would have enough time to complete everything before the exam. If I did not plan, I would not know my progress. During the weekend, I could study more because there was no class.”	LA2, Interview: “I had no plan. I just did questions and learned as much as I could. I did not have a plan of what I wanted to do because I thought I already made it to medical school, so why should I try harder? I did not have a plan to review the materials and did not take a systematic approach.” LA2, Reflective Journal: “I have learned that medicine isn’t something that I can do well without enough focus and with the last-minute work. It is something that I need to plan if I want to achieve my goals. I need to reassess my priorities and think about my future and try to come up with a plan.”
			Learning Approach	HA6, Interview: “When I studied, I tried to understand the reasoning behind the topics, instead of just memorizing them blindly. It helps me remember and apply them better.” HA2, Interview: “I could not memorize without understanding because at the end of the day, I need to understand what I am doing as a doctor and I can’t just be doing it out of memorization. If a patient came to ask me ‘Dr, why did this situation happen?’, I wouldn’t be able to explain it by just memorizing it. That’s why I need to understand it in order to give better care.”	LA5, Interview: “I would skim through lecture notes aimlessly. It was just for my satisfaction knowing that I had read the lecture notes. It was not for absorbing any knowledge. I would read all the content and notes that I had done on the slides without understanding them.”
3	Non-academic	Self-management	Time management	HA3, Reflective Journal: “Since the beginning of Year 1, I have made a daily and monthly schedule so that I can manage my time wisely and will not miss any lecture notes.”	LA1, Reflective Journal: “I buried myself in all sorts of entertainment such as watching movies, eating unhealthy food and sleeping. My attention was drawn to many distractions in life, so I did not spend time on my studies. I spent most of my time going out and having fun to escape from the work that was piled up.” LA5, Interview: “I want to change how I use my time before the midterm examination, especially the arrangement of my activities and the mindset that I can catch up with whatever I have missed easily. That was the biggest mistake I have made in my life. There is no way that I can repeat the foundation module, it is too much for me to cover. I will try to manage my time better.”
			Consistency	HA2, Reflective Journal: “I studied consistently every day. I made sure that I kept up with lectures and did not lag at any point in time. I also practice my clinical skills by clerking patients in the wards and taking a history from them as well as practicing physical examinations with my friends. I believe that studying consistently is very important for pre-clinical medical students.”	LA3, Reflective Journal: “I started with full passion by paying attention in classes and did revisions. As the days went by and the subjects became more confused and difficult, I put the difficult subjects aside and focussed on subjects that I found easy.”
4	Non-academic	Flexibility of Mindset	Exposure of Vulnerability and Support Seeking	HA11, Reflective Journal: “No man is an island as no one can achieve something great all by himself/herself especially when it comes to medicine, which revolves around multidisciplinary teamwork. That is why we learn faster and more efficiently by forming study groups for knowledge sharing and discussions.”	LA1, Interview: “I was afraid of disappointing people around me and they would look down on me. I think for all medical students, we must have a certain degree of achievement in our academics to enrol in medical school. Some of us felt insecure when people knew we had questions and doubts because they might think we are not smart enough. I had this thought so I did not dare to ask for academic support. I did not dare to ask for help from my friends and parents. I did not dare to ask their advice when I had those thoughts.” LA1, Interview: “I felt good that I was not afraid to ask help from people anymore. I was not afraid to ask my juniors who have just attended the lecture whereas I have gone through it twice. I was not afraid that they would look down on me because I am repeating the year. I was more focussed on trying to understand the content better. It was a small step but I was very happy. After multiple attempts of asking different people, I finally understood the lecture.”
			Willingness to Adapt to Changes	HA2, Interview: “At the beginning of the second module, I was lost and did not know how to study because my pre-university studying methods couldn’t work here. I had to change my studying methods and it contributed to my good results in the midterm examination.”	LA1, Reflective Journal: “I remained the same ways of studying which I’ve been using since I was in secondary school. It did not work but I did not bother to refine it.” LA1, Reflective Journal: “I did not want to deal with the challenges and only wanted to escape the responsibilities as a medical student.”
